# Different polymorphisms in HIF-1α may exhibit different effects on cancer risk in Asians: evidence from nearly forty thousand participants

**DOI:** 10.18632/aging.103871

**Published:** 2020-11-04

**Authors:** Yichen Liu, Xiaoqi Zhu, Xiaoyi Zhou, Jingwen Cheng, Xiaoyu Fu, Jingsheng Xu, Yuya Wang, Yueping Zhong, Minjie Chu

**Affiliations:** 1 Department of Epidemiology, School of Public Health, Nantong University, Nantong, Jiangsu, China; 2 Center for Disease Control and Prevention of Nantong, Nantong, Jiangsu, China; 3 Department of Surgery, Affiliated Hospital of Nantong University, Nantong, Jiangsu, China

**Keywords:** *HIF-1α*, polymorphism, cancer, expression, survival

## Abstract

The effect of different SNPs in *HIF-1α* and cancer susceptibility remain indistinct. Here, we evaluated the association between all identified SNPs (rs11549465, rs11549467 and rs2057482) in *HIF-1α* and the overall risk of cancer in all case-control studies published before April 2020. A total of 54 articles including 56 case-control studies were included in this analysis. We found that variant genotypes of rs11549465 and rs11549467 were associated with a significantly increased overall cancer risk. In contrast, the variant T allele of rs2057482 showed a significantly reduced risk of overall cancer. In addition, variant genotypes of the three studied SNPs exhibited a significant association with cancer risk in Asians and specific cancer types. Meanwhile, *HIF-1α* was significantly highly expressed in head and neck squamous cell carcinoma and pancreatic cancer tissues. More importantly, survival analysis indicated that the high expression of *HIF-1α* was associated with a poor survival in patients with lung cancer. These findings further provided evidence that different SNPs in *HIF-1α* may exhibit different effects on overall cancer risk; these effects were ethnicity and type-specific. Further studies with functional evaluations are required to confirm the biological mechanisms underlying the role of *HIF-1α* SNPs in cancer development and progression.

## INTRODUCTION

Cancer is one of the leading causes of death worldwide, according to the latest statistics. There were more than 16.9 million cancer cases in the United States in 2019, and that number is expected to rise to more than 22.1 million by 2030 [1]. In comparison, although China has a lower cancer incidence rate, the cancer-associated mortality in China is 30-40% higher than that in the USA. There is no denying that environment and lifestyle play critical roles in the development of cancer, contributing to about 40% of all cancer cases, compared to the other risk factors [2]. Genetic factors are also considered one of the principal factors of cancer [3], among which, heritability is an important genetic parameter defined as the proportion of phenotypic variance caused by any set of single nucleotide polymorphisms (SNPs) [4]. According to a long-term follow-up study among Nordic twins, the overall heritability of cancer is 33% [5].

Various classic pathways may result in the occurrence of tumorigeneses, such as autophagy [6], angiogenesis [7], and hippo signalling [8]. Besides, numerous studies have indicated that the microenvironment at the centre of the tumour is hypoxic during tumour development [9]. Because of severe hypoxia, cancer cells are characterised by dysplasia as they proliferate, as well as structural and functional abnormalities during angiogenesis [10]. The activation of hypoxia-independent mechanisms of hypoxia-inducible factor (HIF) signalling pathway is a sign of cancer [11]. Hypoxia-inducible factor -1 (HIF-1) is an essential transcription factor that regulates cellular response to hypoxia [12]. Mounting evidence shows that the inhibition of HIF-1 activity can significantly inhibit tumour growth [13]. HIF-1 consists of HIF-1α and HIF-1β subunits [14]. It is known that HIF-1α regulates a series of physiologic cancer pathways, such as cell proliferation, apoptosis, and angiogenesis. Hypoxia can stabilise HIF-1α, inhibit its modifications, and also maintain its transcriptional activity [15].

Moreover, SNPs in *HIF-1α* that modify cancer susceptibility have been studied extensively. Most of these studies focused on three common *HIF-1α* SNPs (rs11549465, rs11549467 and rs2057482). However, the results have been indistinct, for instance, a study previously reported that variant T allele of rs11549465 significantly increases the risk of developing lung cancer [16]. In contrast, another study reported that the same variant exhibits no significant association with lung cancer risk, where the effect value was in the opposite direction relative to the previous study [17]. Meanwhile, although some meta-analyses have been performed to investigate the association between *HIF-1α* SNPs and cancer risk, most of these did not incorporate all previously published research. For example, the meta-analysis performed by Li et al. did not integrate three previously published articles and was flawed, which affects the authenticity and accuracy of the research conclusions [18]. There is currently no collective meta-analysis covering all available SNPs in *HIF-1α* together. Therefore, in this study, we aimed to ascertain the association between the different known *HIF-1α* SNPs (rs11549465, rs11549467 and rs2057482) and cancer susceptibility using a total of 54 previously published articles including 56 case-control studies; the pattern of the effects of these SNPs on cancer risk was also evaluated.

## RESULTS

### Characteristics of the published studies

Following the application of strict screening criteria, 54 articles, including 56 case-control studies with a total of 16,901 cases and 21,836 controls, were ultimately included in the quantitative analysis. General characteristics of the included studies are listed in Supplementary Table 1. Among these 54 articles, 28 had been carried out among Asian populations, and 26, among Caucasian populations. Among the articles that explored the relationships between *HIF-1α* SNPs and cancer risk, 4 focused on 3 SNPs (rs11549465, rs11549467 and rs2057482), and 33 focused on 2 SNPs, and the remaining 17 focused on 1 SNP. The distribution of genotypes and alleles of *HIF-1α* polymorphisms (rs11549465, rs11549467 and rs2057482) for each study is listed in Supplementary Tables 2–4.

### Quantitative synthesis

The variant T allele of rs11549465 was associated with a significantly increased cancer risk (dominant model: OR = 1.18, 95% CI = 1.04-1.34) (Table 1). The variant A allele of rs11549467 was also correlated with a significantly increased cancer risk (dominant model: OR = 1.59, 95% CI = 1.20-2.12) (Table 2). On the contrary, the variant T allele of rs2057482 exhibited a significant association with decreased cancer risk (dominant model: OR = 0.87, 95% CI = 0.80-0.95) (Table 3).

**Table 1:** Summary ORs of the *HIF-1α* rs11549465 polymorphism and cancer risk.

**Variables**	**Studies**	**CT versus CC**				**T versus C**				**Dominant model**		
		**OR(95%CI)**	***P*^a^**	***I ^2^***		**OR (95%CI)**	***P*^a^**	***I ^2^***		**OR(95%CI)**	***P*^a^**	***I ^2^***
Total	51	1.11(0.97-1.28)	<0.001	64.8%		1.24(1.09-1.42)	<0.001	73.8%		1.18(1.04-1.34)	<0.001	67.0%
*Ethnicity*												
Asians	26	1.15(0.98-1.34)	0.006	47.3%		1.25(1.07-1.46)	<0.001	56.9%		1.22(1.05-1.43)	0.001	53.1%
Caucasians	25	1.08(0.85-1.36)	<0.001	74.2%		1.26(1.03-1.56)	<0.001	80.8%		1.14(0.93-1.40)	<0.001	74.7%
*Cancer type*												
breast	8	1.05(0.90-1.23)	0.276	19.4%		1.12(0.98-1.29)	0.096	42.3%		1.09(0.94-1.27)	0.188	30.0%
prostate	7	1.25(0.94-1.67)	<0.001	82.7%		1.25(0.96-1.64)	<0.001	84.4%		1.27(0.95-1.69)	<0.001	83.9%
renal	5	0.80(0.44-1.44)	0.001	77.5%		1.01(0.69-1.48)	0.004	74.0%		0.80(0.43-1.46)	0.001	79.8%
colorectal	5	0.83(0.24-2.83)	0.005	81.4%		0.92(0.37-2.26)	0.019	74.6%		1.28(0.75-2.20)	0.013	68.3%
lung	4	1.19(0.78-1.82)	0.044	63.0%		1.23(0.69-2.20)	<0.001	84.3%		1.23(0.71-2.13)	0.002	79.1%
head and neck	5	1.05(0.68-1.62)	0.135	46.0%		2.18(0.83-5.71)	<0.001	83.2%		1.16(0.77-1.74)	0.325	13.5%
cervical	3	0.98(0.72-1.34)	0.084	59.7%		1.41(0.59-3.35)	<0.001	88.3%		1.32(0.61-2.87)	0.006	80.4%
endometrial	2	1.69(0.18-16.15)	0.003	88.5%		2.12(0.46-9.78)	0.001	90.3%		2.29(0.25-21.11)	0.001	90.1%
hepatocellular	2	0.96(0.17-5.29)	0.021	81.3%		1.14(0.59-2.22)	0.061	71.5%		1.06(0.24-4.68)	0.035	77.4%
pancreatic	2	0.50(0.02-14.02)	0.001	90.3%		1.77(1.24-2.52)	0.349	0.0%		1.39(0.54-3.56)	0.032	78.1%
*System*												
urinary^b^	6	0.80(0.44-1.44)	0.001	77.5%		1.00(0.69-1.48)	0.004	74.0%		0.88(0.54-1.42)	0.001	74.7%
female reproductive^c^	6	1.14(0.68-1.90)	0.011	66.2%		1.47(0.81-2.67)	<0.001	84.8%		1.37(0.75-2.49)	<0.001	77.8%
digestive^d^	16	0.96(0.63-1.46)	<0.001	68.9%		1.31(0.93-1.85)	<0.001	67.4%		1.20(0.91-1.57)	0.005	55.7%

^a^ P for heterogeneity, random-effects model was used when *P* value for heterogeneity test < 0.05; otherwise, fixed-effect model was used

^b^ The urinary system cancer includes renal cancer and bladder cancer

^c^ The female reproductive system cancer includes cervical cancer, endometrial cancer and ovarian cancer

^d^ The digestive system cancer includes colorectal cancer, esophagus cancer, gastric cancer, liver cancer, oral cancer, and pancreatic cancer

**Table 2:** Summary ORs of the *HIF-1α* rs11549467 polymorphism and cancer risk.

**Variables**	**Studies**	**GA versus GG**				**A versus G**				**Dominant model**		
		**OR(95%CI)**	***P*^a^**	***I ^2^***		**OR (95%CI)**	***P*^a^**	***I ^2^***		**OR(95%CI)**	***P*^a^**	***I ^2^***
Total	39	1.51(1.16-1.96)	<0.001	73.2%		1.74(1.28-2.36)	<0.001	84.5%		1.59(1.20-2.12)	<0.001	80.5%
*Ethnicity*												
Asians	21	1.53(1.19-1.97)	<0.001	66.7%		1.54(1.18-2.01)	<0.001	74.4%		1.50(1.15-1.96)	<0.001	72.3%
Caucasians	18	1.34(0.67-2.69)	<0.001	79.0%		2.06(0.91-4.67)	<0.001	89.2%		1.77(0.86-3.65)	<0.001	86.0%
*Cancer type*												
breast	6	1.26(0.95-1.68)	0.112	50.0%		1.29(0.99-1.68)	0.056	60.3%		1.28(0.97-1.70)	0.077	56.1%
lung	4	1.59(1.21-2.10)	0.652	0.0%		1.68(1.03-2.76)	0.042	63.4%		1.80(1.39-2.33)	0.177	39.2%
head and neck	5	2.49(1.06-5.85)	0.009	70.3%		6.08(1.06-34.73)	<0.001	94.7%		5.15(1.26-21.12)	<0.001	90.5%
renal	4	1.51(0.45-5.05)	<0.001	91.7%		1.53(0.60-3.92)	<0.001	89.0%		1.58(0.49-5.04)	<0.001	91.6%
cervical	3	0.78(0.52-1.19)	0.513	0.0%		0.74(0.49-1.10)	0.653	0.0%		0.76(0.50-1.14)	0.578	0.0%
colorectal	3	1.05(0.45-2.45)	0.304	5.5%		1.05(0.45-2.43)	0.307	4.2%		0.91(0.55-1.52)	0.534	0.0%
prostate	3	1.41(0.97-2.07)	0.365	0.7%		1.45(1.00-2.10)	0.330	9.9%		1.44(0.98-2.10)	0.340	7.2%
hepatocellular	2	1.42(0.17-11.54)	<0.001	93.1%		1.34(0.17-10.84)	<0.001	93.5%		1.39(0.16-11.81)	<0.001	93.5%
pancreatic	2	1.61(0.24-10.76)	0.019	81.9%		3.08(1.98-4.78)	0.418	0.0%		3.14(1.99-4.97)	0.098	63.4%
*System*												
urinary^b^	5	1.51(0.45-5.05)	<0.001	91.7%		1.53(0.60-3.92)	<0.001	89.0%		1.36(0.51-3.59)	<0.001	91.0%
female reproductive^c^	5	0.85(0.56-1.27)	0.190	39.8%		0.79(0.53-1.18)	0.200	37.8%		0.82(0.54-1.22)	0.194	39.1%
digestive^d^	13	2.11(1.28-3.46)	<0.001	72.6%		3.15(1.52-6.53)	<0.001	89.7%		2.54(1.39-4.65)	<0.001	85.3%

^a^ P for heterogeneity, random-effects model was used when *P* value for heterogeneity test < 0.05; otherwise, fixed-effect model was used

^b^ The urinary system cancer includes renal cancer and bladder cancer.

^c^ The female reproductive system cancer includes cervical cancer, endometrial cancer and ovarian cancer

^d^ The digestive system cancer includes colorectal cancer, esophagus cancer, gastric cancer, liver cancer, oral cancer, and pancreatic cancer

**Table 3:** Summary ORs of the *HIF-1α* rs2057482 polymorphism and cancer risk.

**Variables**	**Studies**	**CT versus CC**				**T versus C**				**Dominant model**		
		**OR(95%CI)**	***P*^a^**	***I ^2^***		**OR (95%CI)**	***P*^a^**	***I ^2^***		**OR(95%CI)**	***P*^a^**	***I ^2^***
Total	9	0.85(0.72-1.00)	0.006	63.1%		0.91(0.85-0.97)	0.201	27.4%		0.87(0.80-0.95)	0.055	47.4%
*Ethnicity*												
Asians	6	0.80(0.66-0.98)	0.002	74.4%		0.90(0.83-0.97)	0.075	50.1%		0.84(0.71-0.98)	0.018	63.5%
Caucasians	3	1.01(0.78-1.31)	0.836	0.0%		0.93(0.83-1.05)	0.701	0.0%		0.99(0.78-1.27)	0.899	0.0%
*Cancer type*												
multiple myeloma	1	0.94(0.59-1.51)				0.89(0.59-1.34)				0.91(0.58-1.43)		
lung	1	0.92(0.69-1.24)				1.00(0.79-1.28)				0.96(0.72-1.27)		
non-hodgkin lymphoma	1	0.98(0.67-1.44)				1.06(0.77-1.46)				1.02(0.71-1.48)		
colorectal	1	1.15(0.70-1.89)				0.92(0.80-1.04)				1.05(0.64-1.71)		
pancreatic	1	0.45(0.33-0.62)				0.76(0.60-0.96)				0.58(0.44-0.77)		
cervical	1	0.71(0.54-0.92)				0.73(0.59-0.90)				0.69(0.54-0.89)		
prostate	1	0.90(0.72-1.13)				0.86(0.72-1.03)				0.87(0.70-1.08)		
renal	1	0.99(0.78-1.26)				1.05(0.87-1.27)				1.02(0.81-1.29)		
breast	1	0.93(0.78-1.10)				0.95(0.82-1.09)				0.93(0.78-1.10)		

^a^ P for heterogeneity, random-effects model was used when *P* value for heterogeneity test < 0.05; otherwise, fixed-effect model was used

### Stratified analysis of ethnicity and cancer type

**Figure 1:**
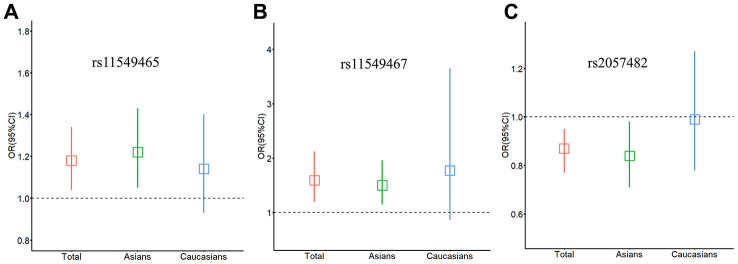
**Relationship between *HIF-1α* SNPs and cancer risk stratified by ethnicity.** (**A**) rs11549465; (**B**) rs11549467; (**C**) rs2057482. Squares represent the ORs and vertical lines represent the corresponding 95% CI.

We evaluated the effect of the 3 SNPs on cancer risk among the subgroups. In the stratified analyses of ethnicity (Figure 1A–1C), the variant T allele of rs11549465 had a significant association with increased risk of cancer among Asian populations (dominant model: OR = 1.22, 95% CI = 1.05-1.43) (Table 1). At the same time, the association between the rs11549467 and the increased risk of cancer was also significant among Asians (dominant model: OR = 1.50, 95% CI = 1.15-1.96) (Table 2). The association between rs2057482 and decreased cancer risk was also significant among Asian populations (dominant model: OR = 0.84, 95% CI = 0.71-0.98) (Table 3). However, none of the 3 SNPs was significantly associated with cancer risk among Caucasians.

When stratified by cancer type, rs11549465 was significantly associated with the risk of pancreatic cancer (T versus C: OR = 1.77, 95% CI = 1.24-2.52) (Table 1). The rs11549467 was associated with the risk of lung cancer (dominant model: OR = 1.80, 95% CI = 1.39-2.33), head and neck cancer (dominant model: OR = 5.15, 95% CI = 1.26-21.12), pancreatic cancer (dominant model: OR = 3.14, 95% CI = 1.99-4.97) and prostate cancer (A versus G: OR = 1.45, 95% CI = 1.00-2.10) (Table 2). Besides, when we classified tumours in different parts of the body by the organ system, the variant A allele of rs11549467 was significantly associated with increased risk of digestive system cancers (dominant model: OR = 2.54, 95% CI = 1.39-4.65) (Table 2).

### Sensitivity analysis and publication bias

**Figure 2:**
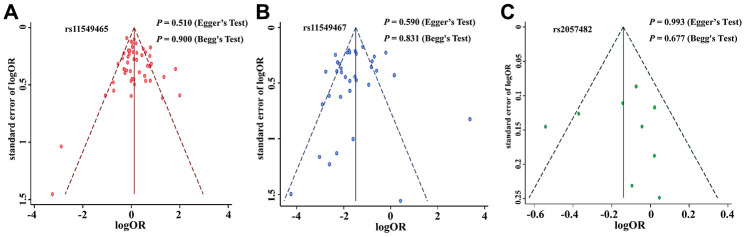
**Funnel plot for publication bias of the *HIF-1α* SNPs and cancer risk.** (**A)**: rs11549465; (**B)**: rs11549467; (**C)**: rs2057482.

We excluded studies that were not in Hardy Weinberg Equilibrium (HWE) to evaluate the stability of the previously acquired results. The results of the 3 SNPs were still statistically significant after omitting the studies that were not in HWE, which confirmed that the obtained results of the meta-analysis were stable and robust. We then utilised the funnel plot, Begg’s test, and Egger’s test to evaluate potential publication bias of the studied literature. The funnel plots were symmetrical in case of all the studied SNPs (Figure 2A–2C). Moreover, Begg’s test and Egger’s test provided further statistical evidence for the absence of publication bias in all the studied SNPs (dominant model: *P* > 0.05).

### HIF-1α expression

**Figure 3:**
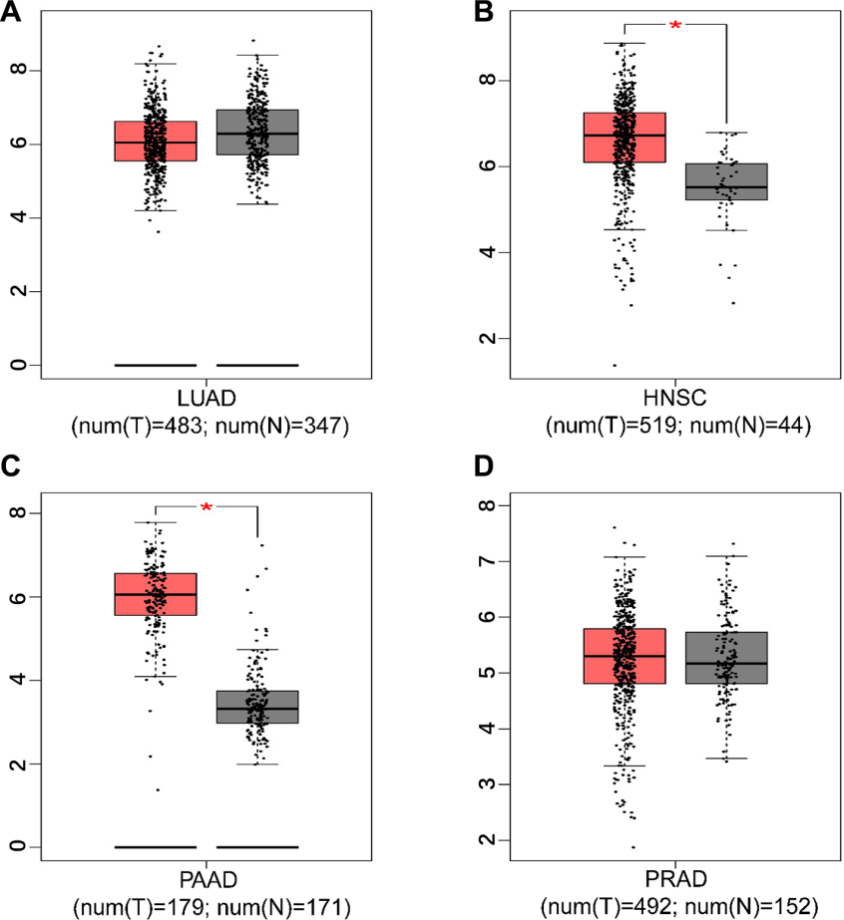
**The expression level of *HIF-1α* in tumor tissues and adjacent non-tumor tissues.** ((**A**) lung adenocarcinoma; (**B**) head and neck squamous cell carcinoma; (**C**) pancreatic adenocarcinoma; (**D**) prostate adenocarcinoma; * *P* < 0.05).

Stratified analysis indicated that *HIF-1α* SNPs (rs11549465, rs11549467, or rs2057482) were mainly associated with the risk of pancreatic, lung, head and neck, and prostate cancers. We then quantified the expression levels of *HIF-1α* in the above four cancers using the GEPIA database. The expression levels of *HIF-1α* was significantly higher in head and neck squamous cell carcinoma (HNSC) and pancreatic adenocarcinoma (PAAD) tissues (*P* < 0.05), as shown in Figure 3. However, we did not observe any significant association for *HIF-1α* expression in lung adenocarcinoma (LUAD) and prostate adenocarcinoma (PRAD) tissues.

### Survival analysis

**Figure 4:**
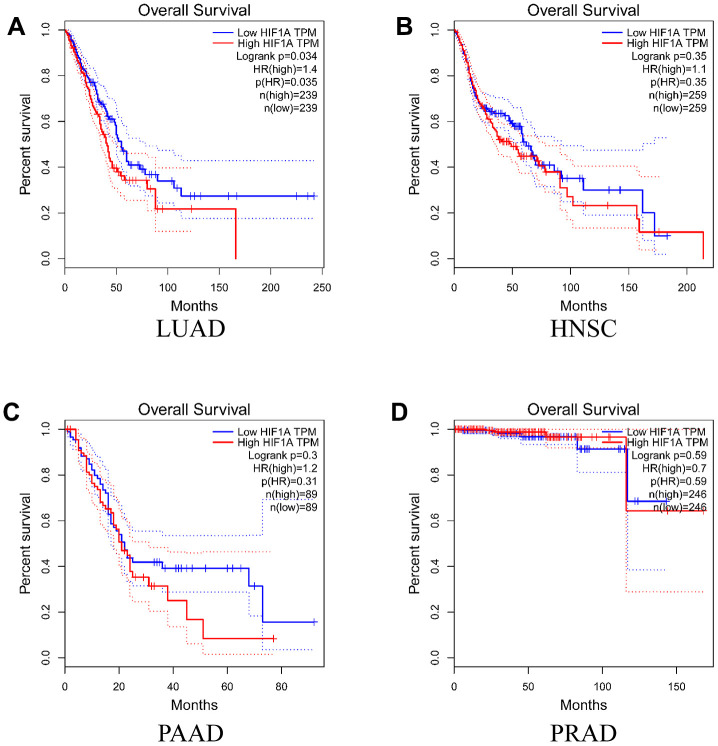
**Overall survival time curves for different expression level of *HIF-1α.*** (**A**) lung adenocarcinoma; (**B**) head and neck squamous cell carcinoma; (**C**) pancreatic adenocarcinoma; (**D**) prostate adenocarcinoma).

To evaluate the function of *HIF-1α* in the survival rate of the above mentioned four cancer types, we conducted Kaplan-Meier analysis according to *HIF-1α* expression and cancer survival based on GEPIA database. As shown in Figure 4, high expression of *HIF-1α* was associated with poor survival in subjects with LUAD (*P* = 0.034). However, *HIF-1α* expression was not associated with the survival of subjects in the other three cancer types.

## DISCUSSION

In this study, we conducted a meta-analysis of 54 articles including 56 case-control studies (up to a total of 16,901 cases and 21,836 controls). We demonstrated that both variant genotypes of rs11549465 and rs11549467 were associated with a significant increase in the overall cancer risk. In contrast, the variant T allele of rs2057482 showed a significantly reduced overall risk of cancer. Moreover, there was evidence of significantly high *HIF-1α* expression in HNSC and PAAD tissues. More importantly, survival analysis indicated that high expression of *HIF-1α* was associated with a poor prognosis in patients with LUAD.

It is well known that one of the characteristics of tumours is the dysregulation of cell proliferation [19]. During the growth of solid tumours, the cells are adequately oxygenated through angiogenesis and glycolytic activation, a process known as the Warburg effect [20]. This effect causes abnormalities in the structure and function of blood vessels, which in turn causes severe hypoxia [21]. HIF-1 is a key transcription factor that regulates oxygen in cells and the entire organism [22]. Many researchers have confirmed that HIF-1A regulates many vital functions, such as lymphatic regeneration [23]. Increasing evidence confirmed that HIF-1α is associated with the development and progression of multiple human cancers [24–26].

Using the GEPIA database, we found that *HIF-1α* was significantly highly expressed in HNSC and PAAD tissues, which hinted its function as an oncogene. A systematic review indicated that high expression of *HIF-1α* is often correlated with adverse clinical characteristics, including the disease stage and differentiation grade, which negatively influences the survival of patients with HNSC [27]. There is a similar study, which reported that oral epithelial dysplastic lesions with increased *HIF-1α* expression are at a high risk of malignant transformation to oral squamous cell carcinoma [28]. In addition, high expression of *HIF-1α*, which is regulated by the LncRNA PVT1/miR-519d-3p axis, promotes glycolysis and pancreatic ductal adenocarcinoma progression [29]. Survival analysis based on GEPIA database indicated that the high expression of *HIF-1α* was associated with a poor prognosis in patients with LUAD, which is consistent with a recent study [30]. It was also reported that proto-oncogene *HIF-1α-*regulated miR-1275 maintains stem cell-like phenotype and promotes the progression of LUAD through the activation of Wnt/β-catenin and Notch signalling pathways [31].

In our study, we interestingly revealed that different SNPs in the same gene might exhibit different effects on cancer risk. Variant genotypes of *HIF-1α* rs11549465 and rs11549467 SNPs were both associated with a significantly increased cancer risk. In contrast, the variant genotype of rs2057482 showed a significantly reduced risk of cancer. Coincidentally, both rs11549465 and rs11549467 are located in exon 12 of *HIF-1α*, and the two SNPs are not in linkage disequilibrium (*r^2^* = 0.005). Based on the DNase I hypersensitive site sequencing (DNase-seq) dataset, we found that exon 12 is within open chromatin regions associated with gene regulatory elements, further ChIP-Seq data from the ENCODE project showed that exon 12 locates in a region which may affect POLR2A transcription factors binding. More importantly, both of them are missense variants. PROVEAN and SIFT (http://provean.jcvi.org/) consistently predict that amino acid substitution resulted from rs11549465 is deleterious (damaging) that may affect protein function, while amino acid substitution resulted from the other SNP rs11549467 is neutral (tolerated). Thus, it is biologically plausible that an amino acid substitution in rs11549465 (Pro>Ser) may lead to the dysfunction of HIF-1α, hence increasing cancer susceptibility. However, the SNP rs2057482 is not located in the exon region; it is in the 3′ UTR region of *HIF-1α.* It is well known that miRNAs can directly mediate post-transcriptional gene silencing through binding to the 3′ UTR of the target gene, which is considered as the canonical mode of miRNA-mediated gene regulation [32, 33]. The target prediction database miRanda was used to identify miRNAs that may target *HIF-1α*, following strict screening criteria (score cutoff ≥ 145, energy cutoff ≤ -15 kcal/mol). Four miRNAs (miR-196a, miR-196b, miR-921 and miR-98) were identified that might bind to 3′ UTR of *HIF-1α.* Among which, a low miR-196b-5p expression is significantly associated with metastases and poor survival in patients with colorectal cancer, while miR-196b-5p inhibition leads to significantly increased colorectal cancer cell migration/invasion and metastases [34]. Also, the expression levels of miR-196b-5p are significantly down-regulated in breast cancer tumour samples compared to the matching normal tissues, while miR-196b-5p over-expression significantly inhibits the proliferation and migration of breast cancer cells [35]. Moreover, it was reported that mir-98-5p is down-regulated in lung cancer cell lines compared to healthy lung epithelial human BES-2B cells, while over-expression of miR-196b-5p inhibits the growth, migration, and invasion in lung cancer cells [36]. The above studies regarding different cancer types indicated that miR-196b and mir-98 might function as a tumour suppressor gene. Considering miRanda database revealed that miR-196b and miR-98 binding to *HIF-1α* is feasible in rs2057482 wild C allele. Thus, it is biologically plausible that the T allele variant of *HIF-1α* SNP rs2057482 might decrease the binding ability of miR-196b and miR-98 to *HIF-1α*, and the increase on miR-196b and miR-98 expressions might of a consequence of this. These miRNAs might hence be involved in the inhibition of cancer development.

It is worth mentioning that when we performed the stratified analysis of ethnicity, both variant genotypes of the studied SNPs exhibited significant association with cancer risk in Asians. However, none of the SNPs exhibited any significant association with cancer risk in Caucasians. There may be two major reasons for these inconsistent results. First, we could not exclude the possibility that genetic heterogeneity between different ethnicities, 28 articles from Asia were included, 85.71% (24/28) of which were from East Asia (China, Japan and Korea), and the genetic background among East Asian populations were relatively similar. Second, different types of cancers may involve random errors. For example, the rs11549465 exhibited no significant association with the risk of renal cancer in stratified analysis, and the effect value was in the opposite direction relative to the overall cancer risk. As expected, 80.0% of the articles that focused on renal cancer (4/5) were in Caucasian populations, which may partly lead to the differences in findings between Caucasians and Asians. Nevertheless, further studies with large sample sizes are warranted to evaluate the relationship between the three studied SNPs and cancer risk in Caucasians.

The advantages of this meta-analysis are apparent. First of all, until now, no study has collectively reported a meta-analysis of all available SNPs in *HIF-1α*. In this study, we extensively reviewed all the available SNPs in *HIF-1α* and screened all possible reports. More importantly, it is encouraging that we arrived at an important conclusion that different SNPs in *HIF-1α* may exhibit different effects on cancer risk. Second, based on the different positions of three SNPs in *HIF-1α*, we explored the possible reasons why the three SNPs exhibit different effects on cancer risk in detail, which may shed light to further biological mechanism studies. Third, using the GEPIA database, we identified that *HIF-1α* might function as an oncogene in a cancer type-specific manner; high *HIF-1α* expression may influence survival in lung cancer patients. However, some limitation also need to be addressed in our study, since we could not extract the original genotyping data for each individual in each study, thus we could not explore the gender effect in the association with cancer types, meanwhile, we could not provide the haplotype analysis for variants rs11549465 and rs11549467.

## CONCLUSION

This study provided new evidence showing that different SNPs in *HIF-1α* exhibit different effects on overall cancer risk. Furthermore, rs11549465, rs11549467 and rs2057482 in *HIF-1α* may modify cancer susceptibility in an ethnicity- and type-specific manner. Further studies with functional evaluations are required to confirm the biological mechanisms underlying the role of *HIF-1α* SNPs in cancer development and progression.

## MATERIALS AND METHODS

### Identification and eligibility criteria of relevant studies

**Figure 5:**
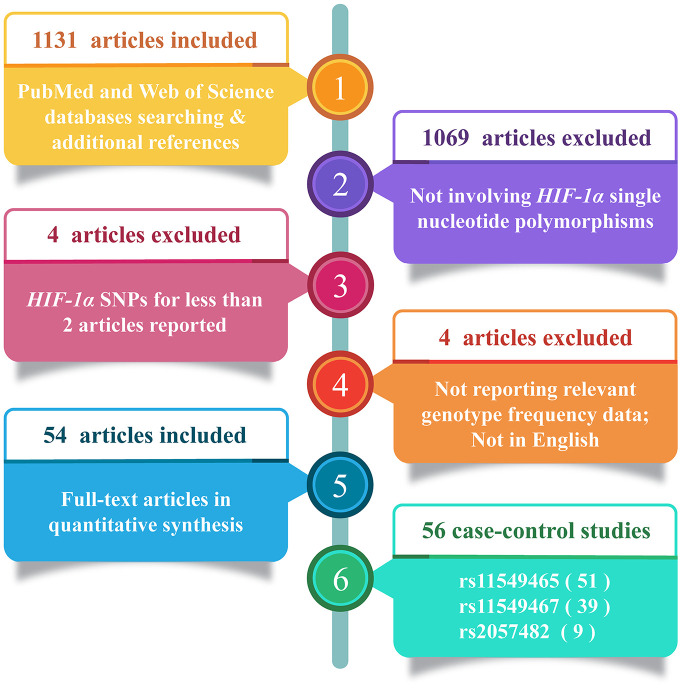
**Flow diagram of the study selection process.**

A comprehensive literature search of research papers published before April 30, 2020, using PubMed and Web of Science databases was performed. We used the following keywords: (“polymorphism”, “variation”, “variant”, or “ mutation”) and (“cancer”, “carcinoma”, “ tumor”, “ tumour”, or “neoplasm”) and (“hif1a”, “*HIF-1A*”, “hif1-a”, “hif1alpha”, “*HIF-1A*lpha”, “hif1-alpha”, “hypoxia inducible factor 1 alpha”, “ hypoxia inducible factor-1alpha”, “hypoxia inducible factor1-alpha”, “hypoxia-inducible factor 1 alpha”, “hypoxia-inducible factor-1alpha” or “hypoxia-inducible factor1-alpha”). The meta-analysis included only full-text articles available in English. In addition, to obtain all eligible publications, the references in the retrieved articles were reviewed. In this meta-analysis, studies meeting the following criteria were included: (1) involving *HIF-1α* polymorphisms and cancer risk; (2) designed as case-control studies; (3) at least two articles for each studied *HIF-1α* SNP; (4) containing available genotype frequencies of *HIF-1α* SNPs (e.g., rs11549465, rs11549467 and rs2057482). The exclusion criteria were as follows: Studies that (1) did not focus on cancer risk; (2) did not study *HIF-1α* SNPs (rs11549465, rs11549467 and rs2057482); (3) did not report relevant genotype frequency data; (4) were not published in English. Finally, 54 articles including 56 case-control studies were included in the meta-analysis (Figure 5).

### Data extraction

Two authors (L.Y. and Z.X.) extracted the data independently. Each article contained the following information: The name of the first author, year of publication, country of origin, ethnicity, type of cancer and numbers of case/control. All disagreements were discussed and resolved, and a consensus was finally reached.

### Functional annotation based on GEPIA

GEPIA (Gene Expression Profiling Interactive Analysis) (http://gepia.cancer-pku.cn) is a novel interactive web server that can be used to explore and analyze the RNA sequencing expression data, based on the 9,736 tumors and 8,587 normal samples from The Cancer Genome Atlas (TCGA) and the Genotype-Tissue Expression (GTEx) projects. More specifically, various customizable functions could be supplied by GEPIA database, including tumor/normal differential expression analysis, profiling according to cancer types or pathological stages, patient survival analysis, similar gene detection, correlation analysis, and dimensionality reduction analysis.

### Statistical analysis

For each study, the odds ratio (OR) and 95% confidence interval (CI) were used to estimate the cancer risk associated with each *HIF-1α* polymorphism. Additionally, the heterogeneity was examined using a chi-square-based Q statistic test, where *P* ≤ 0.05 was considered statistically significant. When heterogeneity between studies was absent, we pooled the results using fixed-effect models. Otherwise, a random-effects model was chosen. Subsequently, we evaluated the risks of the heterozygous genotype relative to the wild-type homozygous genotype and then assessed the risks of the combined heterozygous as well as variant homozygous genotypes relative to the wild-type homozygous genotype. We also assessed the allele model. Besides, we performed a stratified analysis based on ethnicity (divided into Asian and Caucasian), and cancer type. Funnel plot, Begg’s test, and Egger’s test were used to assess publication bias. All analyses were performed using Stata SE version 15.1 software (Stata Corporation, College Station, TX, USA).

## Supplementary Material

Supplementary Tables
