# Supplementary material for: Different polymorphisms in HIF-1α may exhibit different effects on cancer risk in Asians: evidence from nearly forty thousand participants
Source: Aging (Albany NY). 2020 Nov 4;12(21):21329–43. doi: 10.18632/aging.103871 (PMC7695358; doi:10.18632/aging.103871)
Supplement: Supplementary Tables [file aging-12-103871-s001..pdf]

SUPPLEMENTARY TABLES

Supplementary Table 1. Characteristics of literature included in the study.

Number	First Author	Year	Country	Ethnicity	Type of cancer	Case/Control	Genotyped SNPs		
							rs11549465	rs11549467	rs2057482
1	Clifford	2001	UK	Caucasian	renal	48/143	√	√	
2	Tanimoto	2003	Japan	Asian	head and neck	55/110	√	√	
3	KUWAI	2004	Japan	Asian	colorectal	100/100	√		
4	Ollerenshaw	2004	UK	Caucasian	renal	160/162(146/288) [a]	√	√	
5	LING	2005	China	Asian	esophageal	95/104	√		
6	Chau	2005	America	Caucasian	prostate	196/196	√		
7	Fransén	2006	Sweden	Caucasian	colorectal	198/258	√	√	
8	Konac	2007	Turkey	Caucasian	cervical	32/107	√	√	
8	Konac	2007	Turkey	Caucasian	endometrial	21/107	√	√	
8	Konac	2007	Turkey	Caucasian	ovarian	49/107	√	√	
9	Li	2007	America	Caucasian	prostate	1072/1271	√	√	
10	Orr-Urtreger	2007	Israel	Asian	prostate	402/300	√	√	
11	Nadaoka	2008	Japan	Asian	bladder	219/461	√	√	
12	Apaydin	2008	Turkey	Caucasian	breast	102/102	√	√	
13	KIM	2008	Korea	Asian	breast	90/102	√	√	
14	Lee	2008	Korea	Asian	breast	1599/1536	√		√
15	Horrée	2008	Netherlands	Caucasian	endometrial	58/559	√		
16	Jacobs	2008	USA	Caucasian	prostate	1420/1450	√		
17	NAIDU	2009	Malaysia	Asian	breast	410/275	√	√	
18	Li	2009	China	Asian	gastric	87/106	√	√	
19	Konac	2009	Turkey	Caucasian	lung	141/156	√	√	
20	Muñoz-Guerra	2009	Spain	Caucasian	head and neck	74/139	√	√	
21	Chen	2009	China	Asian	head and neck	174/347	√	√	
22	Foley	2009	Ireland	Caucasian	prostate	95/188	√		
23	MORRIS	2009	UK	Caucasian	renal	332/313	√	√	
24	Knechtel	2010	Austria	Caucasian	colorectal	381/2156	√	√	
25	Frank	2010	Germany	Caucasian	colorectal	1768/1794			√
26	HSIAO	2010	China	Asian	hepatocellular	102/347	√	√	
27	Shieh	2010	China	Asian	head and neck	305/96	√	√	
28	Kim	2011	Korea	Asian	cervical	199/214	√	√	
29	KANG	2011	Korea	Asian	colorectal	50/50	√		
30	Xu	2011	China	Asian	glioma	150/150	√		
31	PUTRA	2011	Japan	Asian	lung	83/110	√	√	
32	Wang	2011	China	Asian	pancreatic	263/271	√	√	
33	Zagouri	2012	Greece	Caucasian	breast	113/124	√		
34	KUO	2012	China	Asian	lung	285/300	√	√	
35	Alves	2012	Brazil	Caucasian	head and neck	40/88	√	√	
36	Ruiz-Tovar	2012	Spain	Caucasian	pancreatic	59/152	√	√	
37	Li	2012	China	Asian	prostate	662/716	√	√	√
38	Qin	2012	China	Asian	renal cell	620/623	√	√	√
39	RIBEIRO	2013	Portugal	Caucasian	breast	96/74	√	√	
40	Mera-Menéndez	2013	Spain	Caucasian	glottic	121/154	√	√	
41	Meka	2014	India	Asian	breast	348/320	√		
42	Sharma	2014	India	Asian	breast	200/200	√	√	
43	Fu	2014	China	Asian	cervical	518/553	√	√	√
44	Liu	2014	China	Asian	hepatocellular	157/173	√	√	
45	Fraga	2014	Portugal	Caucasian	prostate	754/736	√		
46	Lessi	2014	Italy	Caucasian	renal	117/1000	√		

47	Ni	2015	China	Asian	Multi [b]	267/275	√	√	
48	YAMAMOTO	2016	Japan	Asian	lung	462/379	√	√	√
49	Peckham-Gregory	2016	USA	Caucasian	non-hodgkin lymphoma	180/528			√
50	Wang	2016	China	Asian	pancreatic	410/490			√
51	Demirel	2017	Turkey	Caucasian	colorectal	92/101	√	√	
52	Shan	2018	China	Asian	breast	560/583		√	
53	Uslu	2018	Turkey	Caucasian	laryngeal	35/35	√		
54	Martina	2018	Czech	Caucasian	multiple myeloma	275/219		√	√

[a] 160/162 for rs11549465; 146/288 for rs11549467

[b] Including multi digestive tract cancers

Supplementary Table 2. Distribution of genotypes of *HIF-1α* rs11549465 polymorphism.

Number	First Author	Type of cancer	Frequency distributions of the genotypes					
			CC_case	CT_case	TT_case	CC_control	CT_control	TT_control
1	Nadaoka	bladder	197	22		419	42	
2	Sharma	breast	152	38	10	149	42	9
3	Meka	breast	245	94	9	229	89	2
4	RIBEIRO	breast	74	21	1	61	9	4
5	Zagouri	breast	98	15	0	107	17	0
6	NAIDU	breast	294	100	16	222	50	3
7	KIM	breast	81	8	1	93	9	0
8	Apaydin	breast	79	21	2	68	29	5
9	Lee	breast	1207	119	6	1245	123	1
10	Fu	cervical	467	49	2	492	60	1
11	Kim	cervical	177	22	0	187	27	0
12	Konac	cervical	10	14	8	68	37	2
13	Fransén	colorectal	167	28	3	213	43	2
14	KUWAI	colorectal	100	0	0	89	11	0
15	KANG	colorectal	38	12		46	4	
16	Demirel	colorectal	62	27	3	81	16	4
17	Knechtel	colorectal	291	77		1773	383	
18	Ni	Multi [a]	219	44	4	241	34	0
19	Horrée	endometrial	50	5	3	463	84	12
20	Konac	endometrial	4	12	5	68	37	2
21	LING	esophageal	84	11	0	93	11	0
22	Li	gastric	83	4	0	93	13	0
23	Xu	glioma	121	27	2	135	14	1
24	Mera-Menéndez	glottic	85	18	15	113	27	8
25	Liu	hepatocellular	152	4	1	162	11	0
26	HSIAO	hepatocellular	94	8	0	334	13	0
27	Tanimoto	head and neck	45	10	0	98	12	0
28	Uslu	laryngeal	28	7	0	28	7	0
29	YAMAMOTO	lung	405	55	2	341	37	1
30	KUO	lung	153	94	38	216	73	11
31	PUTRA	lung	74	9	0	98	12	0
32	Konac	lung	110	31	0	111	43	2
33	Muñoz-Guerra	head and neck	57	6	7	113	27	8
34	Chen	head and neck	163	10	1	334	13	0
35	Alves	head and neck	0	1	39	0	85	3
36	Shieh	head and neck	282	23	0	89	7	0
37	Konac	ovarian	34	14	1	68	37	2

38	Ruiz-Tovar	pancreatic	47	1	11	116	28	8
39	Wang	pancreatic	209	54	0	242	29	0
40	Fraga	prostate	579	164	11	566	156	14
41	Li	prostate	612	48	2	659	57	0
42	Foley	prostate	65	30	0	175	13	0
43	Li	prostate	818	209	14	995	221	18
44	Chau	prostate	161	29	6	179	14	3
45	Jacobs	prostate	1156	252	12	1138	284	28
46	Orr-Urtreger	prostate	287	99	16	217	80	3
47	MORRIS	renal	290	39	3	262	46	5
48	Lessi	renal	82	30	5	808	181	11
49	Qin	renal	572	46	2	578	43	2
50	Ollerenshaw	renal	16	54	90	1	90	71
51	Clifford	renal	42	6	0	110	27	6

[a] Including multi digestive tract cancers

Supplementary Table 3. Distribution of genotypes of *HIF-1α* rs11549467 polymorphism.

Number	First Author	Type of cancer	Frequency distributions of the genotypes					
			GG_case	GA_case	AA_case	GG_control	GA_control	AA_control
1	Nadaoka	bladder	204	15		421	40	
2	Shan	breast	501	55	4	544	37	2
3	Sharma	breast	200	0	0	200	0	0
4	RIBEIRO	breast	96	0	0	74	0	0
5	NAIDU	breast	332	72	6	232	41	2
6	KIM	breast	87	3	0	94	7	1
7	Apaydin	breast	102	0	0	98	4	0
8	Fu	cervical	489	29	0	510	42	1
9	Kim	cervical	187	12	0	200	13	1
10	Konac	cervical	32	0	0	107	0	0
11	Fransén	colorectal	189	9	0	247	9	0
12	Demirel	colorectal	91	1	0	98	3	0
13	Knechtel	colorectal	356	11		2080	76	
14	Ni	Multi [a]	221	41	5	259	16	0
15	Konac	endometrial	21	0	0	107	0	0
16	Li	gastric	74	13	0	100	6	0
17	Mera-Menéndez	glottic	107	4	0	130	9	0
18	Liu	hepatocellular	147	10	0	151	21	1
19	HSIAO	hepatocellular	87	15	0	333	14	0
20	Tanimoto	head and neck	51	4	0	101	9	0
21	YAMAMOTO	lung	407	53	2	343	32	4
22	KUO	lung	150	94	41	215	74	11
23	PUTRA	lung	72	9	2	101	9	0
24	Konac	lung	140	1	0	154	2	0
25	Martina	multiple myeloma	259	15	1	211	7	1
26	Muñoz-Guerra	head and neck	40	21	3	130	9	0
27	Chen	head and neck	153	20	1	333	14	0
28	Alves	head and neck	2	1	37	81	7	0
29	Shieh	head and neck	281	24	0	89	7	0
30	Konac	ovarian	47	2	0	107	0	0
31	Ruiz-Tovar	pancreatic	54	2	3	142	10	0
32	Wang	pancreatic	198	65	0	249	22	0
33	Li	prostate	614	47	1	685	31	0
34	Li	prostate	1053	13	0	1247	17	0

35	Orr-Urtreger	prostate	198	2	0	298	2	0
36	MORRIS	renal	313	10	2	294	15	0
37	Qin	renal	575	45	0	584	39	0
38	Ollerenshaw	renal	65	67	14	239	39	10
39	Clifford	renal	47	1	0	140	4	0

[a] Including multi digestive tract cancers

Supplementary Table 4. Distribution of genotypes of *HIF-1α* rs2057482 polymorphism.

Number	First Author	Type of cancer	Frequency distributions of the genotypes					
			CC_case	CT_case	TT_case	CC_control	CT_control	TT_control
1	Martina	multiple myeloma	225	47	3	176	39	4
2	YAMAMOTO	lung	302	138	22	244	121	14
3	Peckham-Gregory	non-hodgkin lymphoma	125	49	6	369	147	12
4	Wang	pancreatic	301	69	40	302	154	34
5	Fu	cervical	343	150	25	318	197	38
6	Li	prostate	418	212	32	428	241	47
7	Qin	renal	388	196	36	393	201	29
8	Frank	colorectal	32	477	1259	34	441	1319
9	Lee	breast	691	415	44	611	396	41
